# Identification of Prognostic Glycolysis-Related lncRNA Signature in Tumor Immune Microenvironment of Hepatocellular Carcinoma

**DOI:** 10.3389/fmolb.2021.645084

**Published:** 2021-04-22

**Authors:** Yang Bai, Haiping Lin, Jiaqi Chen, Yulian Wu, Shi’an Yu

**Affiliations:** 1 Department of Hepatobiliary and Pancreatic Surgery, Affiliated Jinhua Hospital, Zhejiang University School of Medicine, Jinhua, China; ^2^ Department of Surgery, the Second Affiliated Hospital, Zhejiang University School of Medicine, Hangzhou, China; ^3^ The Affiliated Hospital of Stomatology, School of Stomatology, Zhejiang University School of Medicine, and Key Laboratory of Oral Biomedical Research of Zhejiang Province, Hangzhou, China

**Keywords:** hepatocellular carcinoma, glycolysis, prognostic model, tumor immune environment, immune checkpoint blockade, bioinformatics analysis

## Abstract

**Purpose:** The purpose of this study was to construct a novel risk scoring model with prognostic value that could elucidate tumor immune microenvironment of hepatocellular carcinoma (HCC).

**Samples and methods:** Data were obtained through The Cancer Genome Atlas (TCGA) database. Univariate Cox analysis, least absolute shrinkage and selection operator (LASSO) analysis, and multivariate Cox analysis were carried out to screen for glycolysis-related long noncoding RNAs (lncRNAs) that could provide prognostic value. Finally, we established a risk score model to describe the characteristics of the model and verify its prediction accuracy. The receiver operating characteristic (ROC) curves of 1, 3, and 5 years of overall survival (OS) were depicted with risk score and some clinical features. ESTIMATE algorithm, single-sample gene set enrichment analysis (ssGSEA), and CIBERSORT analysis were employed to reveal the characteristics of tumor immune microenvironment in HCC. The nomogram was drawn by screening indicators with high prognostic accuracy. The correlation of risk signature with immune infiltration and immune checkpoint blockade (ICB) therapy was analyzed. After enrichment of related genes, active behaviors and pathways in high-risk groups were identified and lncRNAs related to poor prognosis were validated *in vitro*. Finally, the impact of MIR4435-2HG upon ICB treatment was uncovered.

**Results:** After screening through multiple steps, four glycolysis-related lncRNAs were obtained. The risk score constructed with the four lncRNAs was found to significantly correlate with prognosis of samples. From the ROC curve of samples with 1, 3, and 5 years of OS, two indicators were identified with high prognostic accuracy and were used to draw a nomogram. Besides, the risk score significantly correlated with immune score, immune-related signature, infiltrating immune cells (i.e. B cells, etc.), and ICB key molecules (i.e. CTLA4,etc.). Gene enrichment analysis indicated that multiple biological behaviors and pathways were active in the high-risk group. *In vitro* validation results showed that MIR4435-2HG was highly expressed in the two cell lines, which had a significant impact on the OS of samples. Finally, we corroborated that MIR4435-2HG had intimate relationship with ICB therapy in hepatocellular carcinoma.

**Conclusion:** We elucidated the crucial role of risk signature in immune cell infiltration and immunotherapy, which might contribute to clinical strategies and clinical outcome prediction of HCC.

## Introduction

Liver cancer is one of the most common malignant tumors with a high rate of metastasis and high mortality (Siegel et al., 2020). With the development of modern medicine, the comprehensive treatment strategy has greatly improved the prognosis of samples with liver cancer (Anwanwan et al., 2020). However, due to the high recurrence rate of liver cancer, the long-term prognosis of samples remains poor (Dufour et al., 2013). Currently, the administrations of immune checkpoint blockade inhibitors have revolutionized antitumor treatment in wide range of cancers. According to preclinical trials, about 20% of samples were observed for objective response, indicating immune checkpoint inhibitors may contribute novel insight into clinical intervention and decision-making of HCC (Cheng et al., 2019). The immune cells function as tumor inhibitor or tumor promoter and may act as important players in the tumor immune microenvironment (TIME) (Lei et al., 2020). Due to characteristics of the immune contexture significantly influencing immune therapy outcome (Zhang et al., 2019), it is worth identifying immune indicators which could predict treatment efficacy and prognosis. At present, the prognosis of samples is typically judged by the grade and stage of tumors (Hu et al., 2019). Tumor mutation burden (TMB), which represents the somatic coding errors such as base substitutions, insertions, or deletions across per million bases, has been termed as a promising indicator for predicting responsiveness to ICB based on numerous researches (Snyder et al., 2014; Rizvi et al., 2015; Chan et al., 2019). Exploring new ways to judge prognosis and clinical outcome is helpful to the survival evaluation and disease treatment of samples.

Long noncoding RNAs (lncRNAs) are similar to mRNA in structure, with a length of more than 200 nucleotides, though they do not have the ability to encode proteins (Kopp and Mendell, 2018). Earlier views believed that lncRNAs were a byproduct of translation and generally did not have a function. At the present time, increasing studies have provided evidence to support that lncRNAs act as a vital regulator in immune response, such as immune activation and antigen release (Carpenter and Fitzgerald, 2018; Denaro et al., 2019). An independent research pointed out that lncRNA GAS5 was downexpressed in HCC tumor compared with normal tissue and interference of lncRNA GAS5 accelerated tumor cell migration by reducing NK cell cytotoxicity (Fang et al., 2019). Likewise, lncRNA TCONS_00019715 could promote antitumor response via harnessing macrophage transformation into the M1 phenotype (Huang et al., 2016). Some studies reported that lncRNAs could serve as novel indicators for disease diagnosis, treatment monitoring, and prognostic prediction in HCC (DiStefano, 2017; Wei et al., 2019). However, with increasing research, it has been found that lncRNAs play an important role in cell growth, differentiation and regulation of gene expression (Schmitt and Chang, 2016). It has been reported that a variety of lncRNAs are stably expressed in HCC tissues and that specific lncRNAs play a significant role in the occurrence and development of HCC (Yuan et al., 2016).

The energy supply of human cells mainly comes from mitochondrial oxidative phosphorylation and glycolysis (Lu et al., 2015). Compared to normal cells, tumor cells choose glycolysis as the main method to supply energy, even under aerobic conditions. This abnormal energy metabolism is an important feature of tumor tissue (Ganapathy-Kanniappan, 2018). In this study, we used a variety of statistical methods to identify glycolysis-related lncRNAs to construct a prognostic risk score model, which provides a novel idea for the TIME characterization and ICB treatment of HCC, contributing to clinical management and decision-making of samples with liver cancer.

## Material and Methods

### Multiomic Data Collection

Gene expression profiling for HCC sample compared with normal tissues were obtained from the TCGA-LIHC project (Supplementary Table S6). The corresponding clinical profiles (Supplementary Table S7) were also downloaded from the TCGA portal as described previously. Four categories of somatic mutation data of HCC samples were downloaded from TCGA database (https://portal.gdc.cancer.gov/). We singled out the mutation data files which were obtained through the “SomaticSniper variant aggregation and masking” platform for subsequent analysis (Supplementary Material in MAF form). We prepared the Mutation Annotation Format (MAF) of somatic variants and implemented the “maftools” (Mayakonda et al., 2018) R package which provides a multitude of analysis modules to perform the visualization process. HCC samples were randomly divided into the training set and verification set at a ratio of 1:1. The clinical characteristics of samples within and across groups were similar. All data were obtained from the TCGA public database, and therefore, there was no need for ethics committee approval.

### Patient Data and Tissue Specimens

Five pairs of HCC tissues and adjacent liver tissues were acquired from samples that underwent surgical resection. Corresponding adjacent tissues were harvested 3 cm from the edges of the tumor lesion. Tissue specimens were immediately put into liquid nitrogen postoperation. The tissues were then stored in a −80°C refrigerator for total RNA extraction. To control the potential confounding factors, all samples were diagnosed with HCC by histopathological examination, while the samples that received chemotherapy or radiotherapy were excluded from the study. All participants have signed the written informed consent form.

### Glycolysis-Related Long Noncoding RNAs

RNA sequencing data of HCC samples were obtained from the TCGA-LIHC project, and noncoding genes were identified according to RefSeq IDs or Ensembl IDs. LncRNAs were retained with reference to NetAffx Annotation files. Glycolysis-related genes were obtained from the gene set “HALLMARK_GLYCOLYSIS” in Molecular Signatures Database (MsigDB) (Liberzon et al., 2015). Pearson correlation analysis was performed on the acquired lncRNAs, as well as glycolysis-related genes. When the correlation coefficient |R| > 0.4 and *p* < 0.005, the two genes were considered to be related. The obtained lncRNA was regarded as glycolysis-related lncRNA. Then, it was visualized using Cytoscape. The processing flow of the data conforms to the relevant policies of NIH TCGA human subject protection.

### Prognostic Risk Score Calculation

Using the training set, we conducted a univariate Cox proportional hazard regression analysis, LASSO regression analysis, and two-step multivariate Cox proportional hazard regression analysis on the glycolysis-related lncRNAs. Finally, we selected four glycolysis-related lncRNAs for incorporation into the risk score. The expression of lncRNAs between normal and cancer tissues was compared. The regression coefficient β of multivariate Cox regression model and lncRNA expression were used to construct risk score formula as follows:

Risk score=β⁡lncRNA1×LncRNA1 Expression+β⁡lncRNA2×lncRNA2 Expression+⋯+β⁡lncRNA n×lncRNA n Expression.

### Prognostic Characteristics of Risk Score

Using the training set, validation set, and all samples, we sorted the samples according to the size of the risk score. The samples were divided into high- or low-risk groups depending on the average risk score. Additionally, we drew the lncRNA expression heat map, risk score distribution map, and risk score and survival relationship map. The Kaplan–Meier method was utilized to draw the survival curve and ROC curve of high- and low-risk samples. In order to determine whether the risk score is an independent prognostic factor, the univariate and multivariate Cox regression analysis was conducted on the risk score and some clinical indicators.

### Nomograph Drawing

In order to construct a quantitative scoring system for prognostic evaluation of HCC samples, a ROC curve was drawn with risk score and partial clinical features. Furthermore, the appropriate indicators were selected to construct a nomogram. Subsequently, we analyzed the calibration curve which showed the prognostic value of as-constructed nomogram.

### Enrichment Analysis of Gene Set Enrichment Analysis

We utilized the “h.all.v7.2. symbols.gmt [cancer hallmarks]” and “c2. cp.kegg.v7.2. symbols.gmt [Curated]” gene sets from the MsigDB of the GSEA (version 4.0) to analyze the risk score and explore the possible cellular pathways.

### Assessment of Correlation of Risk Score With Tumor Immune Environment Characterization

To distinguish TIME difference between low-/high-risk subgroups, we employed several analyses as follows. R package “ESTIMATE” was utilized to estimate tumor purity and the extent and level of infiltrating cells (stromal cell and immune cell), which reflected the characteristics of tumor immune microenvironment. Subsequently, single-sample gene set enrichment analysis was conducted via the R package “GSEAbase” to elucidate the enrichment of 29 immune function–related gene sets. The subpopulation of 22 immune cells in each tumor sample was explored through immune cell subtype identification by using CIBERSORT (https://cibersort.stanford.edu/). Furthermore, we compared the expression levels of 46 immune checkpoint blockade–related genes, (i.e. CD274, etc.) between low-risk samples and high-risk samples.

### Assessment of Correlation of Signature With Tumor Immune Infiltration

Immune infiltration information contains each tumor sample’s immune cell fraction (i.e. B cells, CD4+T-cells, CD8+T-cells, dendritic cells, macrophages, and neutrophils), which were obtained from Tumor Immune Estimation Resource (TIMER) (https://cistrome.shinyapps.io/timer/). The correlation of tumor immune cell infiltrating with prognostic risk signature was analyzed to explore whether risk signature could act as a novel and reliable indicator in tumor of immune microenvironment of HCC.

### Assessment of Role of Risk Signature in Immune Checkpoint Blockade Treatment

Based on reported researches, immune checkpoint blockade key targets expression level might be closely associated with clinical outcome of immune checkpoint inhibitors (Goodman et al., 2017). Herein, we selected six key genes of immune checkpoint blockade–related genes: programmed death ligand 1 (PD-L1, namely CD274), programmed death ligand 2 (PD-L2, namely PDCD1LG2), programmed death 1 (PD-1, namely PDCD1), cytotoxic T-lymphocyte antigen 4 (CTLA-4), indoleamine 2,3-dioxygenase 1 (IDO1), and T-cell immunoglobulin domain and mucin domain-containing molecule-3 (TIM-3, namely HAVCR2) in HCC (Kim et al., 2017; Nishino et al., 2017; Zhai et al., 2018). To investigate the potential role of lncRNA-based signature in ICB therapy of HCC, we correlated risk signature with expression level of six immune checkpoint blockade key targets.

### Cell Lines and Culture

One human normal hepatocyte cell line (HL-7702) and two human HCC cell lines (HepG2 and MHCC97H) were cultured in Dulbecco’s Modified Eagle Medium (DMEM, Gibco, United States) containing 10% fetal bovine serum (FBS, Gibco, United States) in a humidified atmosphere at 37°C, containing 5% CO_2_.

### Quantitative Real-Time PCR

For specific qPCR steps, please refer to previous literature (Zhang et al., 2016). The primer sequences used in this study were as follows: MIR4435-2HG forward, 5′-GAC​TCT​CCT​ACT​GGT​GCT​TGG​T-3′ and reverse 5′-CAC​TGC​CTG​GTG​AGC​CTG​TT-3′; glyceraldehyde-3-phosphate dehydrogenase (GAPDH) forward, 5′-CAG​GAG​GCA​TTG​CTG​ATG​AT-3′ and reverse 5′-GAA​GGC​TGG​GGC​TCA​TTT-3′. The relative gene expression levels were calculated by normalizing to GAPDH.

### Statistical Analysis

Statistical analysis was performed by R software (version 4.0.2; R Foundation). Comparisons between multiple groups were analyzed using a one-way analysis of variance (ANOVA) and comparisons between the two groups were analyzed by Student’s t-test. Construction of the glycolysis-related lncRNA co-expression network was carried out with Cytoscape software (version 3.7.2; The Cytoscape Consortium). *p* < 0.05 was considered as significant difference.

## Results

### Multiple lncRNAs Are Associated With Glycolysis-Related Genes

Overall, 14,142 lncRNAs were identified using the TCGA-LIHC database, and glycolysis-related genes were identified using the Molecular Signatures Database. To identify glycolysis-related lncRNAs, Pearson’s correlation test was performed. lncRNAs with Pearson’s correlation coefficient with an absolute value of >0.4 and *p* < 0.005 were set for further analysis. Finally, 1,699 glycolysis-related lncRNAs were obtained (Supplementary Table S1).

### LASSO Regression Analysis Was Able to Accurately Identify Long Noncoding RNAs With Prognostic Value

**FIGURE 1:**
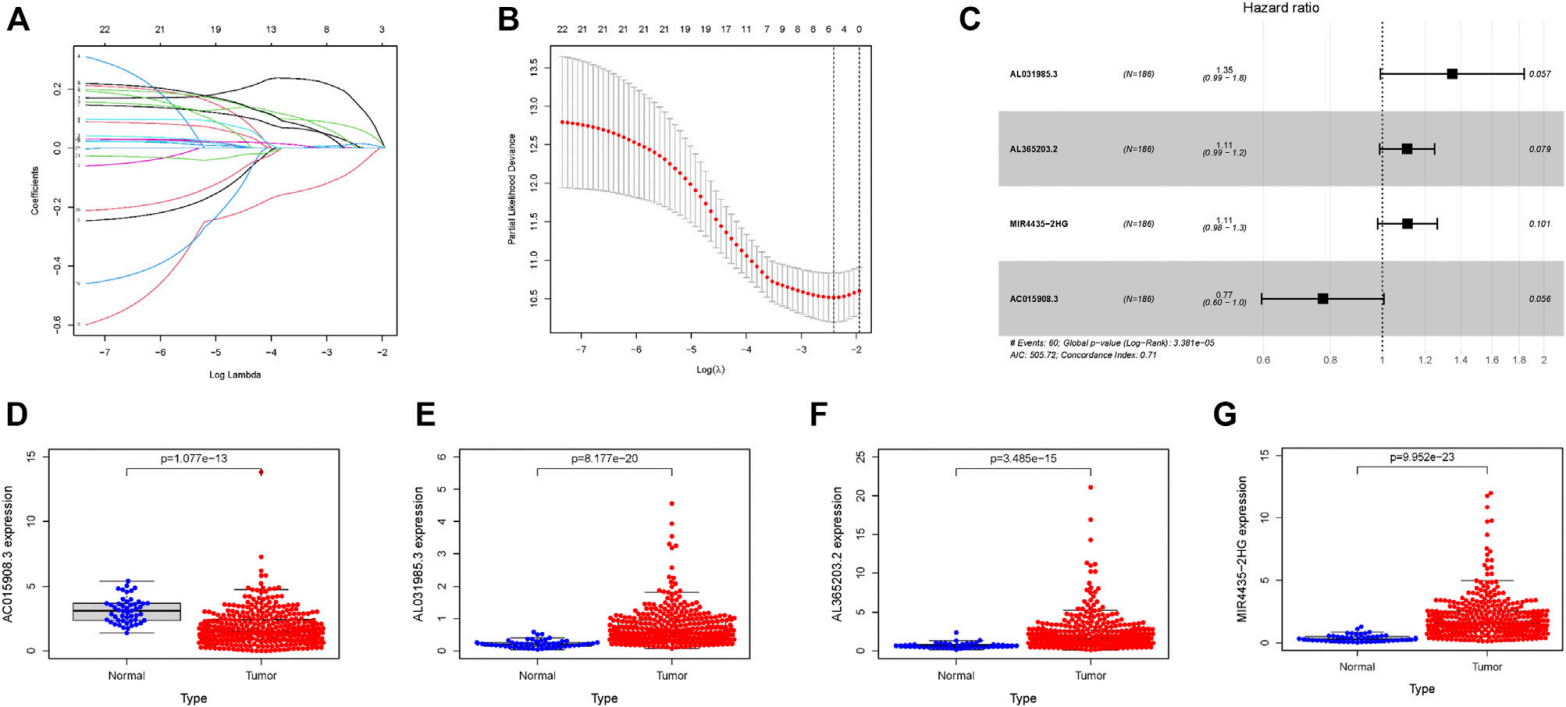
Four glycolysis-related lncRNAs with prognostic value in the training set. **(A)** Plots for LASSO expression coefficients of 22 glycolysis-related lncRNAs. **(B)** Cross-validation plot for the penalty term. **(C)** Relationship between four glycolysis-related lncRNAs and prognosis of HCC patients **(D–G)** Expression of four glycolysis-related lncRNAs in tumors and normal tissues; the data come from TCGA database, where all *p* values < 0.05.

According to the process shown in Supplementary Figure S1, 377 HCC samples were obtained using the TCGA database, and seven samples with incomplete information were excluded from the study. In total, 370 samples were selected for further research. The basic clinicopathological information of samples is shown in Table 1. A detailed description was recorded in Supplementary Table S7. A total of 22 glycolysis-related lncRNAs were identified using univariate Cox analysis, with results shown in Supplementary Table S4. In order to exclude the overfitting, LASSO regression analysis was conducted on 22 lncRNAs, and a total of five glycolysis-related lncRNAs were identified. The screening process and results are shown in Figures 1A,B, and Supplementary Table S5. These five lncRNAs were analyzed using a two-step multivariate Cox regression analysis. Finally, four glycolysis-related lncRNAs were found to be associated with prognosis of HCC samples (Figure 1C). Among them, AL031985.3, AL365203.2, and MIR4435-2HG were found to be poor prognostic factors (hazard ratio, HR > 1), and their expression was upregulated in HCC samples. On the other hand, AC015908.3 was a protective factor (HR < 1), and its expression was found to be decreased in HCC samples. The results are shown in Figures 1D–G and Table 2. Four lncRNAs were used to construct the co-expression network, the results of which are shown in Supplementary Figures S1B,C. According to expression of lncRNAs and multivariate Cox regression coefficient, the prognosis risk score of glycolysis-related lncRNAs was calculated as follows (0.299987 × AL031985.3 expression) + (0.105369 × AL365203.2 expression) + (0.107428 × MIR4435-2HG expression) − (0.25568×AC015908.3 expression). Samples were equally and randomly divided into training set and verification set, including 186 cases in the training set and 184 cases in the verification set. The results of random grouping are shown in Supplementary Tables S2, S3.

**TABLE 1:** Baseline data of all HCC samples.

Characteristic	Type	n	Proportion (%)
Age	≤65	235	62.33
	>65	141	37.40
	Unknown	1	0.27
Gender	Female	122	32.36
	Male	255	67.64
Grade	G1-2	235	62.33
	G3-4	137	36.34
	Unknown	5	1.33
Stage	Stage I–II	262	69.50
	Stage III–IV	91	24.14
	Unknown	24	6.37
T Stage	T1–2	280	74.27
	T3–4	94	24.93
	Unknown	3	0.80
M Stage	M0	272	72.15
	M1	4	1.06
	Unknown	101	26.79
N stage	N0	257	68.17
	N1	4	1.06
	Unknown	116	30.77

**TABLE 2:** Multivariate Cox results of lncRNAs based on TCGA-LIHC data.

Id	Coef	HR	HR.95 L	HR.95H	*p* value
AL031985.3	0.299,987	1.349,841	0.991,382	1.837,909	0.05678
AL365203.2	0.105,369	1.111,121	0.987,831	1.249,799	0.079101
“MIR4435-2HG”	0.107,428	1.113,411	0.979,232	1.265,977	0.101,078
AC015908.3	-0.25568	0.774,388	0.595,609	1.006829	0.056244

### The Risk Score Is Significantly Related to Patient Prognosis

**FIGURE 2:**
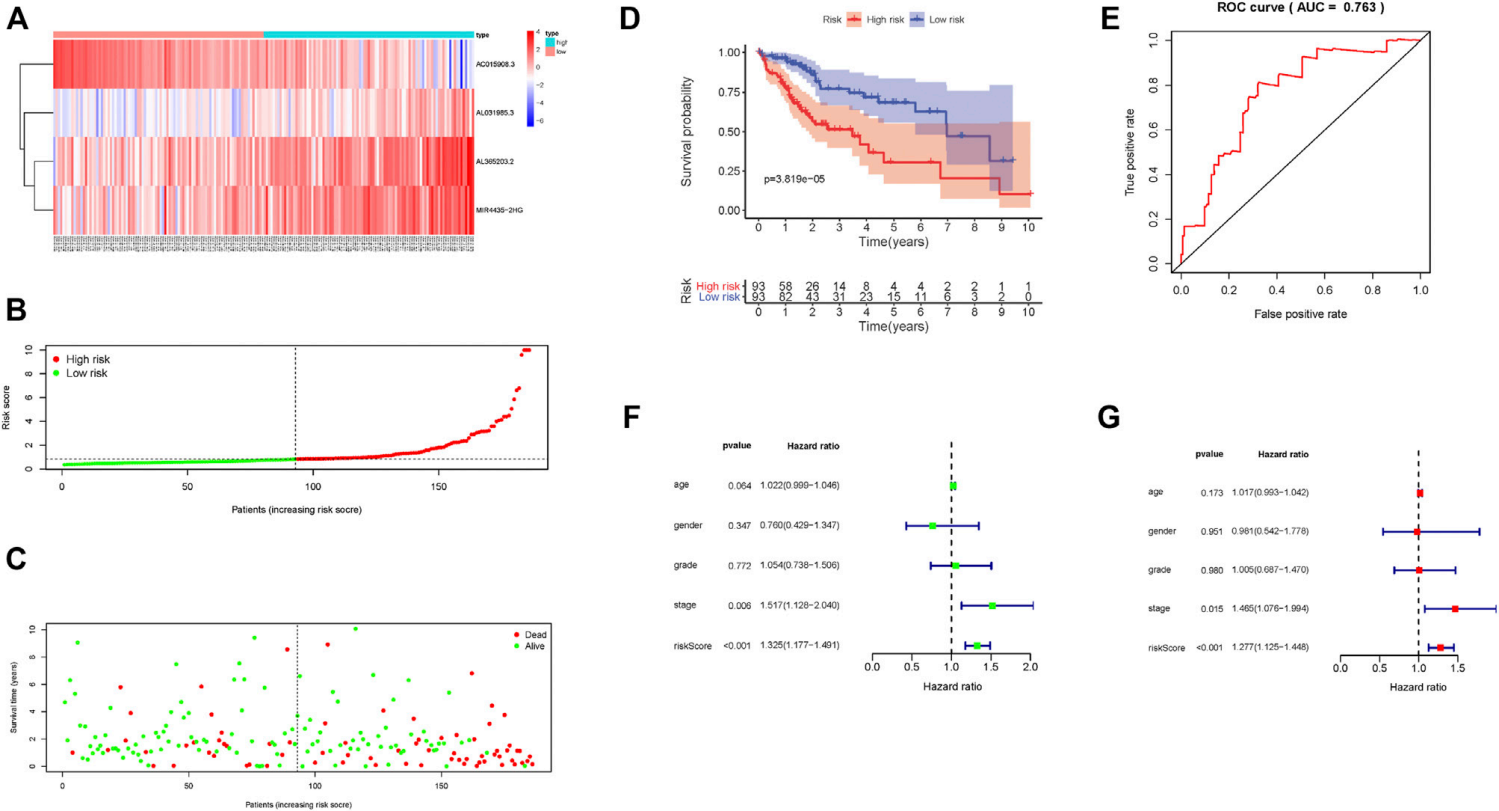
Prognostic risk score characteristics of glycolysis-associated lncRNAs in the training set. **(A)** Heat map of the expression of four lncRNAs in HCC samples. The color from green to red indicates a trend from low expression to high expression. **(B)**. Distribution of risk scores for HCC samples. **(C)** The relationship between survival time and status of HCC samples and risk score. **(D)** Kaplan–Meier survival curve of samples in high- and low-risk groups. **(E)** ROC curve of risk score in samples with HCC. **(F)** Univariate Cox regression analysis of clinical features and risk score in HCC samples. **(G)** Multivariate Cox regression analysis of clinical features and risk score in HCC samples.

According to this scoring system, the prognostic risk score of each patient was calculated and samples were arranged from left to right according to their score level. The heat map distribution of four lncRNAs is shown in Figure 2A. With increasing risk score, the number of surviving samples decreased and the amounts of dead samples increased. The prognosis of samples in the low-risk group was significantly better than that in the high-risk group (Figures 2B,C). The Kaplan–Meier survival curve shows that the 5-year survival rate of samples in the low-risk group is significantly higher than that in the high-risk group (Figure 2D, *p* = 3.819*e* − 05). Moreover, these four lncRNAs were used to construct a prognosis scoring system with high accuracy (Figure 2E, AUC = 0.763). Consistent with these results, univariate and multivariate Cox regression analysis showed that the increased risk score indicates the higher the risk score, the poorer the prognosis (Figures 2F,G).

### Validation of Prognostic Risk Score

**FIGURE 3:**
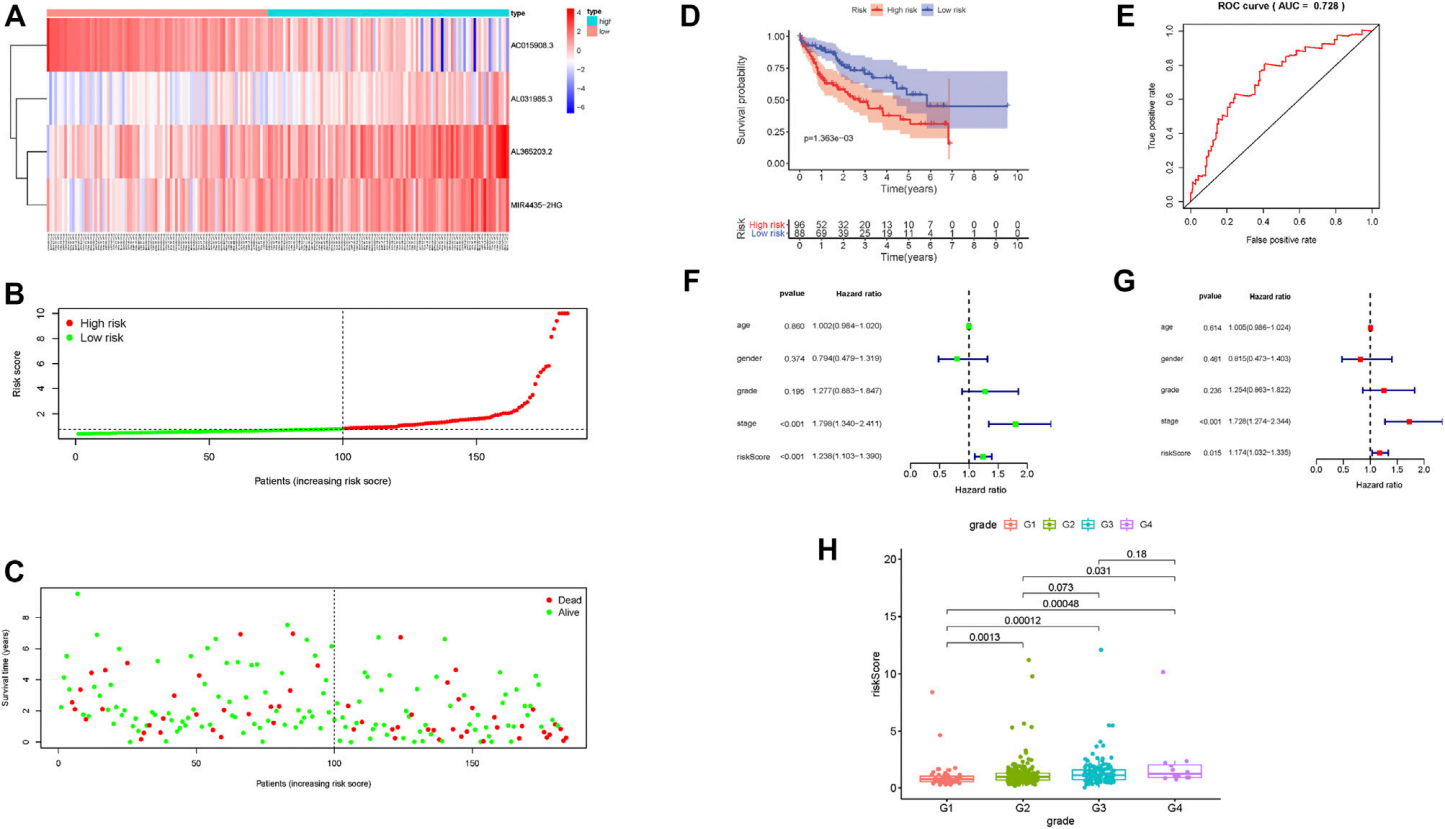
Prognostic risk score characteristics of glycolysis-related lncRNA in the validation set. **(A–C)** Heat map of the expression of four glycolysis-related lncRNAs in HCC samples, distribution map of risk score, relationship map of survival status and risk score. **(D–E)** Kaplan–Meier survival curve and ROC curve of high- and low-risk group. **(F–G)**. Univariate and multivariate Cox regression analysis of clinical features and risk score in HCC samples. **(H)** Relationship between tumor grade and risk score; risk score significantly increased for advanced grade cases.

The risk scoring system was validated using an internal validation set, as well as all samples. The four lncRNAs had similar distributions in the heat map, as well as risk score distribution (Figures 3A,B; Supplementary Figure S2A,B). The higher the risk score, the fewer samples survived and the more deaths that occurred (Figure 3C; Supplementary Figure S2C). The 5-year survival rate in the low-risk group was significantly higher (Figure 3D; Supplementary Figure S2D). The risk scoring system in the validation set, as well as overall samples, has the same degree of predictive accuracy as the training set (Figure 3E; Supplementary Figure S2E). Consistent with results from the training set, a risk score can be used as an independent prognostic factor to judge patient prognosis. The higher the risk score, the worse the prognosis (Figures 3F,G; Supplementary Figures S2F,G), the more serious the tumor grade (Figure 3H).

### Close Correlation of Risk Score With Tumor Immune Environment Characterization of Hepatocellular Carcinoma

To further uncover the potential role of prognostic risk score in TIME of HCC, we investigated the relationship between risk score and immune-related score (calculated with the R package “ESTIMATE”), immune signature (via ssGSEA analysis) and Tumorinitiating cell subtypes and level (assessed by CIBERSORT method), and the 46 immune checkpoint blockade–related genes expression level.

**FIGURE 4:**
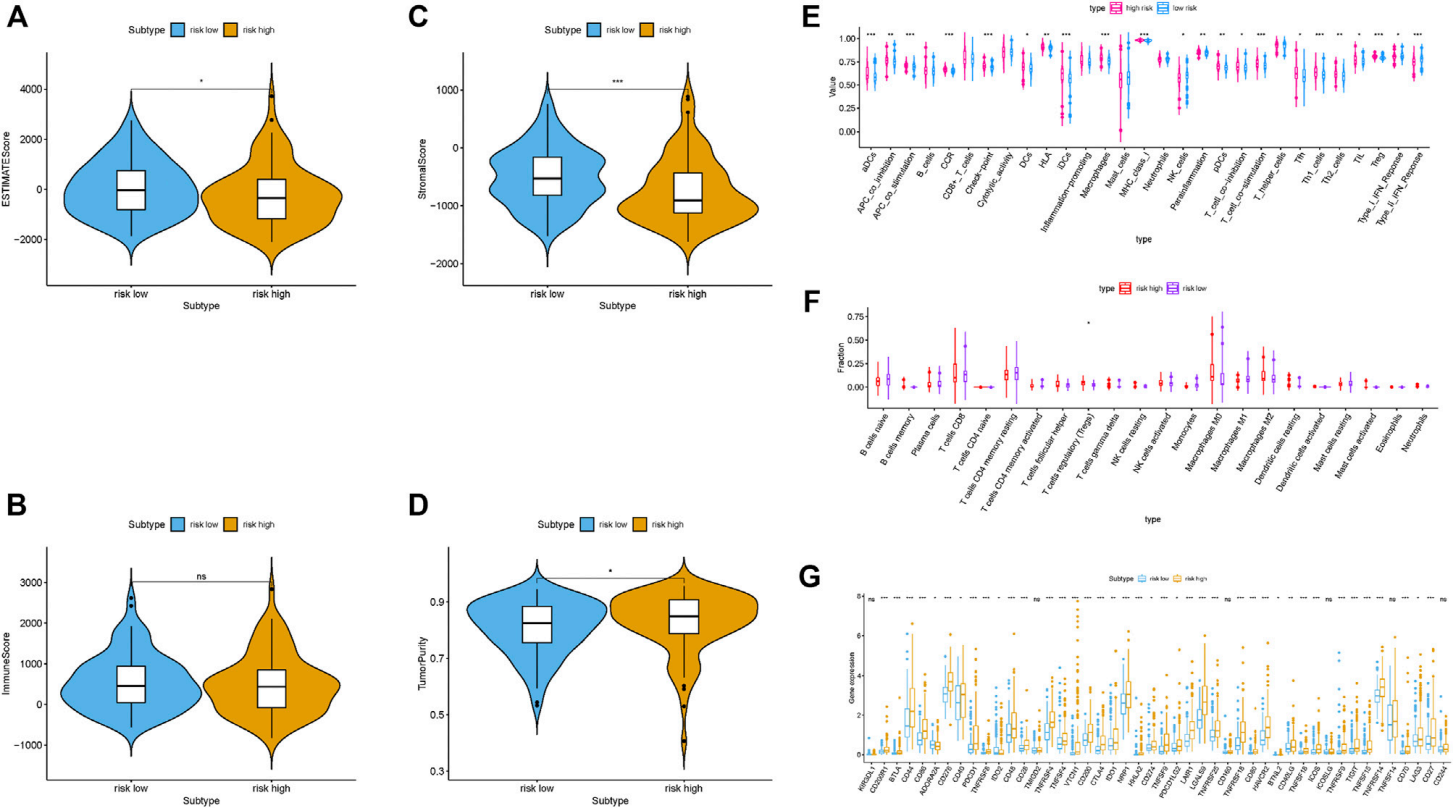
Correlation of prognostic risk score with TIME characterization of HCC **(A–D)** Comparison of estimate score, stromal score, immune score, and tumor purity between these two subtypes. **(E)** Distinction of enrichment of immune-related signatures between low- and high-risk groups. **(F)** Difference of infiltrating immune cell subpopulations and levels between low-/high-risk groups. **(G)** Comparison of 46 immune checkpoint blockade–related genes expression levels in two risk score subgroups.

These results indicated that samples with low risk had a higher estimate score, stromal score, immune score but lower tumor purity compared with high-risk samples (Figures 4A–D). Then, we examined whether there was distinction of immune signatures between groups low/high risk. From the ssGSEA results, we found that the infiltrating levels of aDCs, DCs, iDCs, macrophages, pDCs, Tfh, Th1 cells, Th2 cells, and Tregs were remarkably elevated and some immune signatures (i.e. APC costimulation, checkpoint, parainflammation, HLA molecule, IFN response type II, and MCH class I) were significantly activated with the increased risk score (Figure 4E; Supplementary Figure S3A). Supplementary Figure S3B shows each patient immune-related signature with corresponding immune-related scores in groups low/high risk. The CIBERSORT analysis results pointed out that the more the fraction of regulatory T cells, the higher the risk score (Figure 4F). Further correlation analysis presented that 40 of 46 (i.e. CD274, IDO1, etc.) immune check blockade–related genes expression levels were significantly different between two risk groups (Figure 4G). These results suggested that lncRNA-based risk signature may contribute a novel insight into TIME feature and immune response of HCC.

### The Predictive Power of Risk Score was Significantly Better Compared to Other Clinical Characteristics

**FIGURE 5:**
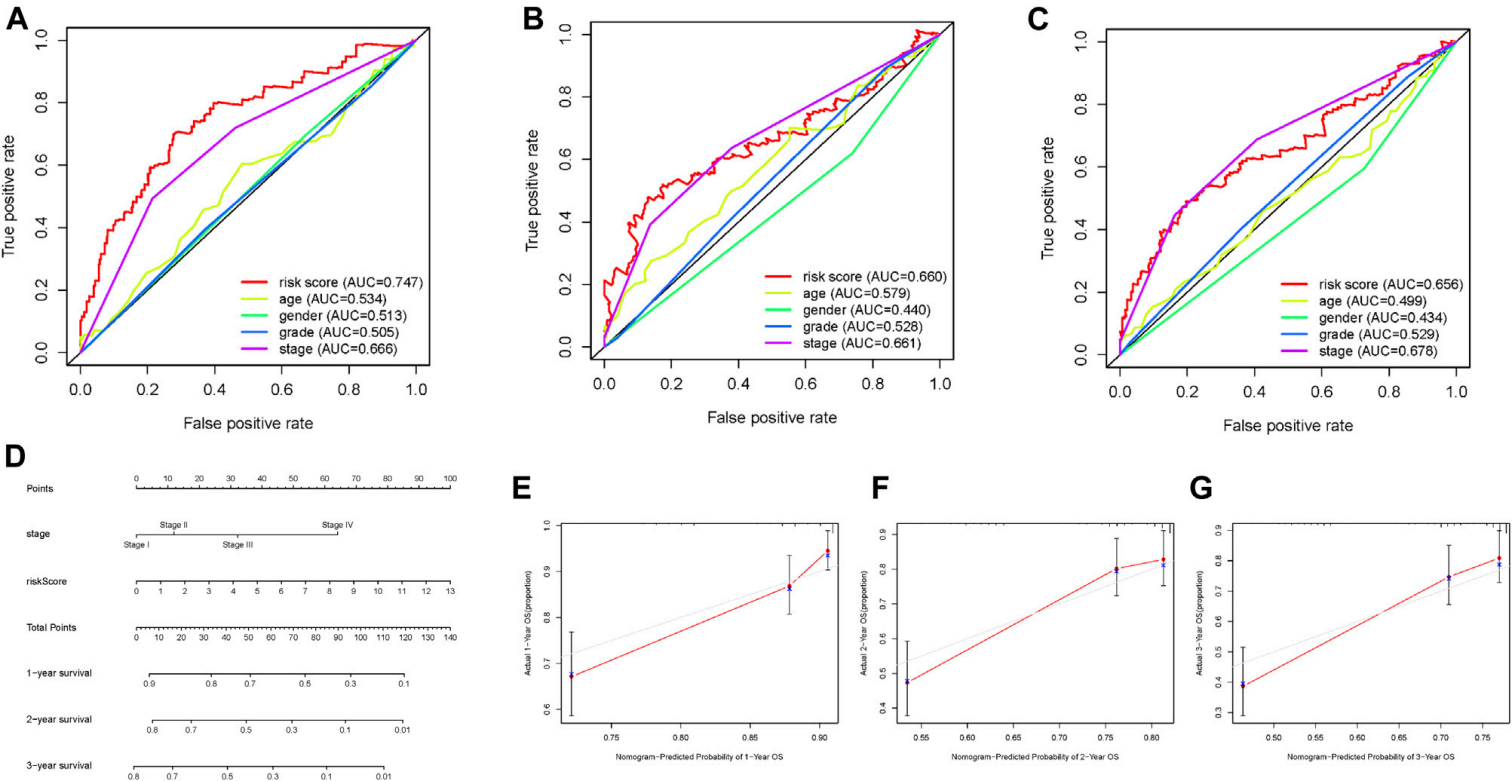
Screening prognostic indicators and nomogram. **(A–C)** ROC curve of 1-, 2-, and 3-year OS for multiple prognostic indicators of HCC samples. **(D)** The nomogram was drawn using tumor grade and risk score. **(E)** One‐year nomogram calibration curves of entire TCGA cohort. **(F)** Two‐year nomogram calibration curves of entire TCGA cohort. **(G)** Three‐year nomogram calibration curves of entire TCGA cohort.

The prognostic risk score, combined with age, gender, and tumor grade and stage, were used to draw ROC survival curve. The results indicated that compared to other clinical traits, the glycolysis-related lncRNA prognostic risk scoring system was more accurate at predicting the 1-, 3-, and 5-year survival rate of HCC samples (Figures 5A–C, AUC = 0.747, 0.660, and 0.656, respectively). The prognostic factors with AUC >0.6 were identified in ROC curve, and the nomogram was drawn. The results are shown in Figure 5D. The 1-, 3-, and 5-year survival rates were calculated quantitatively according to the tumor stage and risk score. We corroborated that our nomograph had great prognostic predictive performance of 1-, 2-, and 3-year survival time by employing calibrate curves (Figures 5E–G).

To validate whether lncRNA risk signature remained with excellent prognostic predictive performance in different clinicopathological subgroups, furthermore, we performed a stratification analysis. Regardless of young or old, the risk signature could further distinguish low-risk group and high-risk group with significantly distinct survival time (Supplementary Figures S5A,B). Likewise, risk signature presented powerful prognosis prediction ability for samples in grade 1–2 or 3–4 (Supplementary Figures S5C,D), early stage or late stage (Supplementary Figures S5E,F), T status one to two or 3–4 (Supplementary Figures S5G,H), N0 status (Supplementary Figure S5I), M0 status (Supplementary Figures S5J) ,and male gender (Supplementary Figure S5K). We found that *p*-value was 0.081, however, female samples’ survival time shortened with the increase of risk score (Supplementary Figure S5L). These results suggested that it can be an outstanding predictor in samples with HCC.

### Risk Score Affects the Results of Gene Enrichment

Hallmark enrichment analysis indicated that apoptosis and glycolysis were active in high-risk group, while being silent in the low-risk group. Additionally, multiple pathways, including IL/STAT5 and NOTCH, were active in the high-risk group and silent in the low-risk group (Supplementary Figure S4A). Finally, Kyoto Encyclopedia of Genes and Genomes (KEGG) enrichment analysis suggested that bladder cancer and colorectal cancer were active in the high-risk group but silent in the low-risk group (Supplementary Figure S4B).

### Correlation of Risk Signature With Infiltrating Immune Cell and Immune Checkpoint Blockade Key Molecules

**FIGURE 6:**
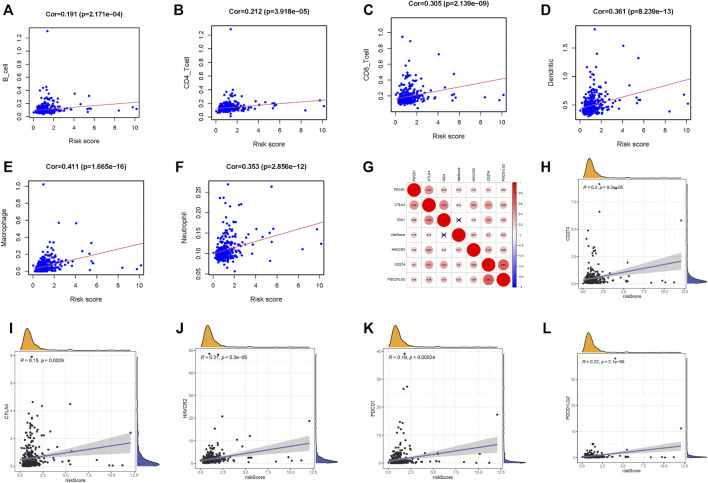
Correlation between tumor immune infiltration and this immune‐related lncRNA signature. **(A)** Association between this signature and B cells. **(B)** Association between this signature and CD4^+^ T cells. **(C)** Association between this signature and CD8^+^ T cells. **(D)** Association between this signature and dendritic cells. **(E)** Association between this signature and macrophages cells. **(F)** Association between this signature and neutrophil cells. **(G)** Association analyses between immune checkpoint inhibitors CD274, PDCD1, PDCD1LG2, CTLA4, HAVCR2, and IDO1 and lncRNAs signature. **(H)** Association between our risk model and CD274. **(I)** Association between our risk model and CTLA4. **(J)** Association between our risk model and HAVCR2. **(K)** Association between our risk model and PDCD1. **(L)** Association between our risk model and PDCD1LG2.

To further explore the influence of lncRNA-based signature upon TIME of HCC, we analyzed correlation of risk signature with immune cell infiltration type and level. We observed that the risk signature significantly correlated with infiltrating B cells (*r* = 0.191; *p* = 2.171*e* − 04), infiltrating CD4+T cells (*r* = 0.212; *p* = 3.918*e* − 05), infiltrating CD8+T cells (*r* = 0.305; *p* = 2.139*e* − 09), infiltrating dendritic cells (*r* = 0.361; *p* = 8.239*e* − 13), infiltrating macrophages (*r* = 0.411; *p* = 1.665*e* − 16), and infiltrating neutrophils (*r* = 0.353; *p* = 2.856*e* − 12; Figures 6A–F). These results suggested that prognostic risk signature was closely correlated with immune infiltration in HCC.

Next, we singled out six key immune checkpoint inhibitor genes (PDCD1, CD274, PDCD1LG2, CTLA‐4, HAVCR2, and IDO1) for further research (Vidyasagar, 2015; Chen et al., 2018; Bejani and Ghatee, 2020). We performed the correlation analysis of ICB key gene expression with risk signature to investigate the potential role of signature in the ICB therapy of HCC (Figure 6G). The analysis result pointed out that risk signature had close relationship with CD274 (*r* = 0.2; *p* = 9.2*e* − 05), CTLA4 (*r* = 0.15; *p* = 0.0029), HAVCR2 (*r* = 0.21; *p* = 5.3*e* − 05), PDCD1 (*r* = 0.19; *p* = 0.00024), and PDCD1LG2 (*r* = 0.22; *p* = 2.1*e* − 05; Figures 6H–L), indicating risk signature might exert a nonnegligible player in ICB treatment outcome prediction in HCC.

### High Expression of MIR4435-2HG in Hepatocellular Carcinoma Suggests Poor Prognosis

**FIGURE 7:**
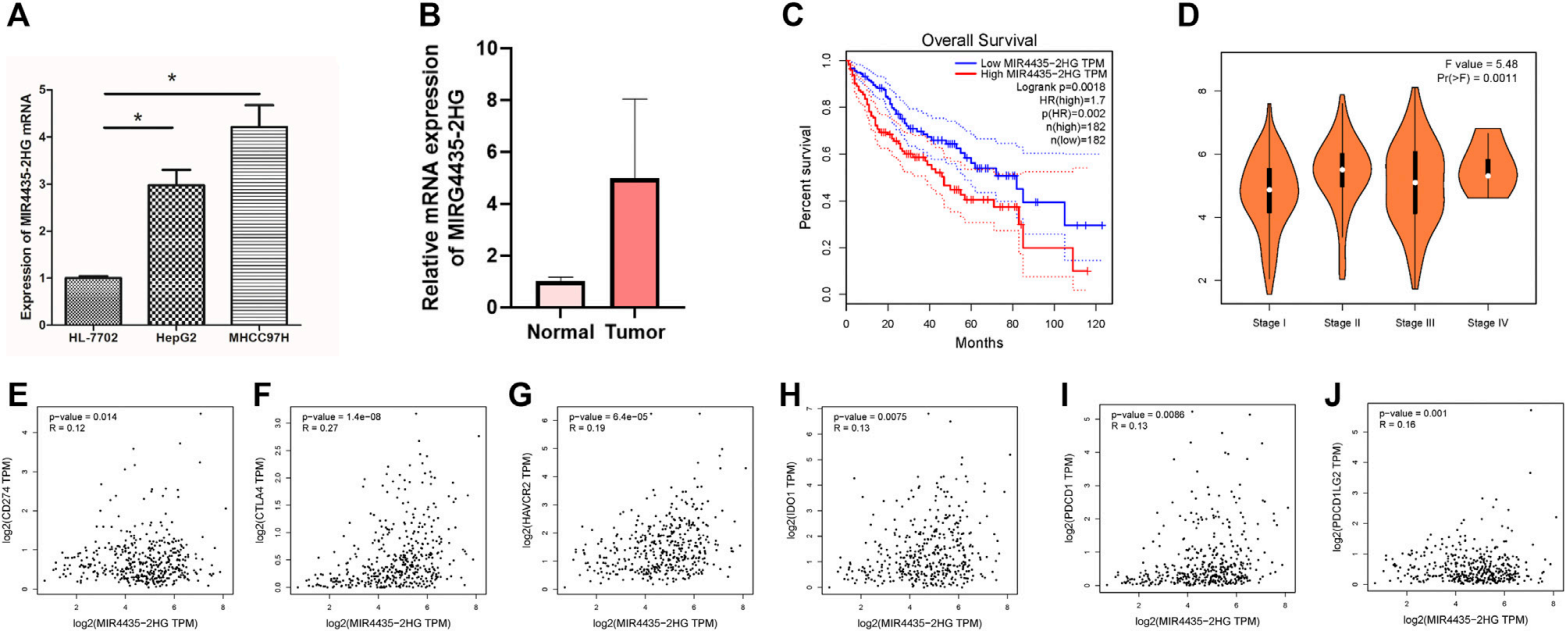
High expression of MIR4435-2HG indicates poor prognosis. **(A)**. qPCR results indicate that MIR4435-2HG is highly expressed in HepG2 and MHCC97H cell lines, *p < 0.05. Each experiment is repeated at least three times. **(B)** qPCR results indicate that MIR4435-2HG is highly expressed in tumor tissue. **(C)** The prognosis of samples with high or low expression of MIR4435-2HG is significantly different. **(D)** The expression of MIR4435-2HG had significant difference between major pathological stages. **(E)** The mRNA expression between MIR4435-2HG and CD274 had more similar pattern in HCC and normal tissues. **(F)** The mRNA expression between MIR4435-2HG and CTLA4 had more similar pattern in HCC and normal tissues. **(G)** The mRNA expression between MIR4435-2HG and HAVCR2 had more similar pattern in HCC and normal tissues. **(H)** The mRNA expression between MIR4435-2HG and IDO1 had more similar pattern in HCC and normal tissues. **(I)** The mRNA expression between MIR4435-2HG and PDCD1 had more similar pattern in HCC and normal tissues. **(J)** The mRNA expression between MIR4435-2HG and PDCD1LG2 had more similar pattern in HCC and normal tissues.

We evaluated the expression of MIR4435-2HG in cell lines and tissues. The results showed that in comparison to normal liver cell lines, the expression of MIR4435-2HG in hepatoma cell lines was significantly increased (Figure 7A, *p* < 0.05). Likewise, MIR4435-2HG was upregulated in tumor tissue relative to normal samples. Limited by number of samples, we observed no statistical difference (Figure 7B). Consistent with the results of *in vitro* experiments, the OS of samples with low expression of MIR4435-2HG was significantly longer than that of samples with high expression (Figure 7C, *p* = 0.0018), suggesting that MIR4435-2HG is a poor prognostic factor for HCC samples. The expression level analysis among major clinical stages shown that MIR4435-2HG expressed significantly differently among distinct clinicopathological stages (Figures 7D, F value = 5.48 and *p* = 0.0011).

### MIR4435-2HG Correlates With Immune Checkpoint Blockade Therapy Key Genes in Hepatocellular Carcinoma

Then we analyzed the correlation between the MIR4435-2HG and ICB-related key genes to elucidate the impact of MIR4435-2HG on the ICB therapy of HCC. The results presented that MIR4435-2HG was significantly positively correlated to CD274 (*r* = 0.12; *p* = 0.014), CTLA4 (*r* = 0.27; *p* = 1.4*e* − 08), HAVCR2 (*r* = 0.19; *p* = 6.4*e* − 05), IDO1 (*r* = 0.13; *p* = 0.0075), PDCD1 (*r* = 0.13; *p* = 0.0086), and PDCD1LG2 (*r* = 0.16; *p* = 0.001; Figures 7E–J), suggesting MIR4435-2HG may be a novel and potential target in ICB treatment in HCC.

### Role of MIR4435-2HG in Tumor Immune Environment Characterization

**FIGURE 8:**
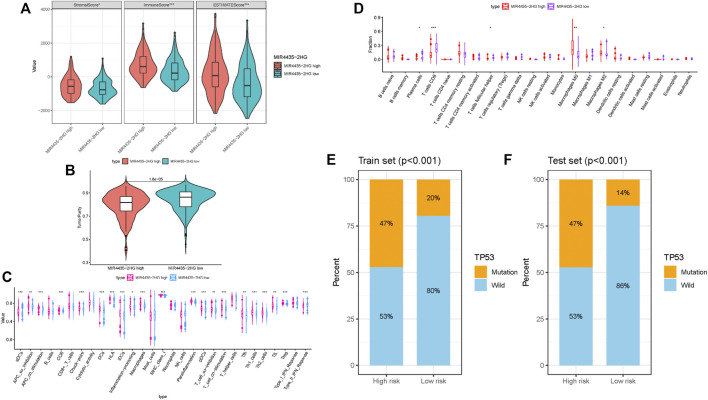
Correlation of MIR4435-2HG in TIME characterization. **(A–B)** Comparison of the immune score (ESTIMATE algorithm) between MIR4435-2HG low/high groups. **(C)** Difference of immune-related signatures between low and high MIR4435-2HG subgroups. **(D)** Distinction of infiltrating immune cell subpopulations and levels between low/high MIR4435-2HG groups. **(E)** Proportion of mutation of TP53 in both low-/high-risk groups form the training set. **(F)** Proportion of mutation of TP53 in both low-/high-risk groups form the testing set.

To further examine whether MIR4435-2HG can act as immune indicators, we performed the correlation analysis of MIR4435-2HG expression level with immune infiltration. HCC samples were classified into high/low MIR4435-2HG subtypes based on the median MIR4435-2HG expression value. ESTIMATE results indicated that samples with higher MIR4435-2HG expression had a significant higher stromal score, immune score, and ESTIMATE score but lower tumor purity relative to samples in high MIR4435-2HG group (Figures 8A,B). Subsequently, we identified difference of enrichment in immune-related signatures between two different subgroups. The subjects in MIR4435-2HG high group remarked as high infiltration of aDCs, DCs, iDCs, pDCs, macrophages, Tfh, Th1 cells, Th2 cells, and Tregs and enrichment of T cell costimulation, APC costimulation, CCR, checkpoint, HLA, inflammation promoting, parainflammation, and class I MHC, which suggested immune-activated phenotype (Figure 8C). The CIBERSORT result presented that expression level of MIR4435-2HG was positively correlated with M0 and M2 macrophage infiltration, whereas negatively correlated with plasma cells, CD8 T cells, and Tfhs (Figure 8D). In summary, these results pointed out that MIR4435-2HG may serve as a key indicator in TIME characterization and immunological reaction in HCC.

### Correlation of Mutation of TP53 With Risk Score

Based on previous research (Ruan et al., 2016), CTSB played a pivotal role in HCC initiation and progression. According to results of somatic mutation data, TP53 was the genes with highest mutation frequency (Supplementary Figure S6A). Thus, we proposed to uncover the role of gene mutation in risk score and analyzed the proportion of mutation gene in both low- and high-risk groups. We observed that mutation of TP53 was significantly correlated with risk score (Figures 8E,F; Supplementary Figure S6B; training set, testing set, and whole cohort, respectively), whereas mutation of CTSB was similar between low- and high-risk groups (Supplementary Figure S6C). These results indicated that mutation of TP53 may contribute to HCC development.

## Discussion

The pathogenesis of HCC is very complex as it involves cell cycle and apoptosis, transcriptional regulation disorder (Lin et al., 2014), and energy metabolism abnormalities (Hsu et al., 2015). LncRNAs affect tumorigenesis and development in many ways, including regulating cell proliferation and migration (Shen et al., 2019), influencing epigenetic regulation (Miao et al., 2019) and regulating energy metabolism rate-limiting enzymes. Glycolysis is an inefficient method of energy production, but this process produces a reduction equivalent (Terabe et al., 2019) and biosynthetic substrate necessary for tumor cell proliferation (Liang et al., 2019). In this study, we obtained clinical and transcriptomic data of HCC from the TCGA database and successively applied univariate Cox analysis, LASSO analysis, and two-step multivariate Cox analysis to identify glycolysis-related lncRNAs. Additionally, abnormal energy metabolism and lncRNAs were combined to construct a risk score model with prognostic value. The model was verified across different groups so that the prognostic judgment of HCC could be quantified and specific and provides guidance for survival prediction of samples.

When selecting specific variables to build a model, there is often overfitting (Dawes et al., 2019). This problem often occurs when there are too many variables. With regard to human genes, only 2% can encode proteins, and 98% of them are noncoding sequences, which constitute a complex regulatory network (Boon et al., 2016). In our study, we observe that there are still 22 lncRNAs that are related to the prognosis of samples after screening by univariate COX analysis, and excessive lncRNAs involved in constructing can cause the risk scoring model to lead to overfitting. An important method to solve overfitting is regularization (Bejani and Ghatee, 2020). LASSO regression constructs a penalty function and adds L1 regularization after the loss function to obtain a more accurate model with fewer variables (Vidyasagar, 2015). After LASSO regression analysis of 22 lncRNAs, only five were found to be related to patient prognosis. Even after two-step multivariate Cox regression, only one lncRNA was identified. The final remaining four lncRNAs indicated high accuracy in the validation set, as well as overall prognosis for samples.

The ROC curves of OS of samples with liver cancer were constructed by combining several clinical characteristics of samples with a prognostic risk score. Indicators with AUC >0.6 were selected to draw a nomogram, which made the judgment of survival rate of samples with liver cancer visualized and more specific. From our results, we are able to see that the risk score based on glycolysis-related lncRNA construction shows high accuracy in predicting the survival rate of samples. The reason is that abnormal energy metabolism plays an important role in metabolomics and epigenetics of liver tumors, and glycolysis-related pathways are significantly related to survival and prognosis of samples (Chen et al., 2018). Furthermore, 90% of energy in normal tissues comes from tricarboxylic acid cycle in mitochondria (Anderson et al., 2018), while more than 50% of the energy depends on glycolysis, which is known as the “Warburg effect” (Pascale et al., 2020). At present, it is believed that the main mechanisms of Warburg effect include mitochondrial dysfunction (Riera Leal et al., 2020), tumor adaptation (Ždralević et al., 2018), microenvironment changes (Sun et al., 2018), oncogene (Banks, 2013), and related signal pathway disorders. According to the results of GSEA enrichment analysis, we found that Notch, p53, Wnt, and other signaling pathways are active in the high-risk group whether we use the Hallmark dataset or KEGG dataset. These pathways are closely related to the recurrence of liver cancer (Invalidcitation, 2018). In addition, we found that glycolysis is active in the high-risk group in the hallmark dataset, which is consistent with our results.

According to published works, we observed that more and more researches focusing on TIME have revealed the potential implication of lncRNAs upon infiltrating immune cells. Peng Lirong et al. reported that LncRNA MIAT was significantly correlated with immune cell infiltration and may exert an important player in the immune escape of HCC (Peng et al., 2020). The study of Ji Jie et al. demonstrated that Lnc-Tim3 was involved in the survival of the exhausted CD8+T cells and facilitating CD8+T exhaustion (Ji et al., 2018). Consequently, we speculated that the subtype of infiltrating immune cells had close connection with lncRNAs. Herein, we corroborated that lncRNA-based risk signature was significantly correlated with immune cell infiltration, (i.e. macrophages, dendritic cells, neutrophils, B cells, CD4+T cells, and CD8+T cells). ESTIMATE results presented that risk score was negatively correlated with estimate score, stromal score, and immune score but positively with tumor purity, suggesting risk signature could serve as a novel immune indicator in HCC. Besides, ssGSEA analysis pointed out that the infiltrating immune cells (i.e. DCs, macrophages, Th1 cells, and Tregs) were significantly increased and immune signatures (i.e. APC costimulation, checkpoint, parainflammation, IFN response type II, and MCH class I) were remarkably activated when risk score elevated. Finally, CIBERSORT algorithm results showed that risk score elevated when the fraction of regulatory T cells increased, indicating that as-constructed signature works through regulating Tregs infiltration and might have an undeniable role in tumor immune microenvironment of HCC. The immune-activated condition in the high-risk group was associated with high ICB-relevant genes expression, suggesting samples in with low risk score might respond to immunotherapy.

With the emergence of immune checkpoint blockade (ICB) treatment, immune checkpoint inhibitors have considerably transformed clinical decision-making in cancer oncology (Pitt et al., 2016; Llovet et al., 2018; Salik et al., 2020). Immune-checkpoint blockade treatment has contributed a novel insight into clinical management in samples with HCC(Ng et al., 2020). Nevertheless, HCC samples obtained relatively few benefits from ICB therapy and less than one in three samples were observed for objective response to immune checkpoint inhibitors treatment (Liu et al., 2020). Such biomolecules as immune checkpoint blockade–related gene expression level and tumor mutational burden were unable to accurately predict clinical outcome of ICB treatment. It is therefore urgent to identify indicators that can precisely forecast responsiveness to ICB treatment for further individualized treatment and advance precision immunotherapy (Nishino et al., 2017; Ng et al., 2020; Mushtaq et al., 2018). Recently, accumulating evidences have supported that numerous lncRNAs possess key roles in regulating immunity, such as immune cell infiltration, antigen presentation, and so on (Carpenter and Fitzgerald, 2018; Denaro et al., 2019). In this study, the correlation analysis showed that PDCD1, CD274, PDCD1LG2, CTLA-4, IDO1, and HAVCR2 were coexpressed. Furthermore, our risk signature was significantly associated with the ICB treatment key target genes (i.e. PDCD1LG2, PDCD1, CD274, HAVCR2, and CTLA4), and the expression level of immune checkpoint blockade–related genes (i.e. IDO1 and TIGHT) increased significantly with increased risk scores. Due to no ICB treatment dataset in HCC cohort, we were unable to explore the correlation between risk score and ICB immunotherapy response. These findings indicated that our signature may possess the ability to predict clinical outcome of ICB therapy in HCC samples.

It has been reported that MIR4435-2HG is associated with prognosis of HCC (Kong et al., 2019). Overexpression of MIR4435-2HG can promote proliferation of HCC cells, which is consistent with our experimental results. However, previous literature has only described this phenomenon. MIR4435-2HG expression was significantly positively associated with ICB immunotherapy key genes (i.e. CD274, CTLA4, HAVCR2, IDO1, PDCD1LG2, and PDCD1). We also demonstrated that MIR4435-2HG expression had close relationship with high infiltration of immune cells (i.e. macrophages) in HCC. These findings indicated that high MIR4435-2HG expression level was associated with a poor prognosis that could facilitate immune evasion and immunotherapy resistance. Our results first linked the mechanism of MIR4435-2HG with immune infiltration and immunotherapy, which provides a new rationale for further research. However, our experiment lacks verification results of clinical samples and only obtains clinical information from the database in order to verify expression of MIR4435-2HG, which is a limitation in our experiment.

## Conclusion

In our study, the LASSO regression method helped identify glycolysis-related lncRNAs to construct a risk score model. This model can quantitatively and accurately judge the prognosis of HCC samples. Moreover, as-constructed lncRNAs signature was significantly correlated to not only immune cell infiltration but also responsiveness to ICB treatment key genes in HCC. Conclusively, this research provided a promising avenue to facilitate the individualized survival prediction and reveal landscape of tumor immune environment of HCC, further contributing valuable clinical applications in HCC ICB therapy. Notwithstanding, our findings should be validated in further researches which explore HCC tumorigenesis and progression mechanisms and the implication of these 4 glycolysis-related lncRNAs.

## Data Availability

Publicly available datasets were analyzed in this study. This data can be found here: https://portal.gdc.cancer.gov/repository?facetTab=files&filters=%7B%22op%22%3A%22and%22%2C%22content%22%3A%5B%7B%22op%22%3A%22in%22%2C%22content%22%3A%7B%22field%22%3A%22cases.primary_site%22%2C%22value%22%3A%5B%22liver%20and%20intrahepatic%20bile%20ducts%22%5D%7D%7D%2C%7B%22op%22%3A%22in%22%2C%22content%22%3A%7B%22field%22%3A%22cases.project.program.name%22%2C%22value%22%3A%5B%22TCGA%22%5D%7D%7D%2C%7B%22op%22%3A%22in%22%2C%22content%22%3A%7B%22field%22%3A%22cases.project.project_id%22%2C%22value%22%3A%5B%22TCGA-LIHC%22%5D%7D%7D%2C%7B%22op%22%3A%22in%22%2C%22content%22%3A%7B%22field%22%3A%22files.data_category%22%2C%22value%22%3A%5B%22transcriptome%20profiling%22%5D%7D%7D%2C%7B%22op%22%3A%22in%22%2C%22content%22%3A%7B%22field%22%3A%22files.data_type%22%2C%22value%22%3A%5B%22Gene%20Expression%20Quantification%22%5D%7D%7D%5D%7D.

## Abbreviations

DMEM, Dulbecco's minimum essential media; GSEA, gene set enrichment analysis; HCC, hepatocellular carcinoma; HR, hazard ratio; KEGG, kyoto encyclopedia of genes and genomes; lncRNAs, long noncoding RNAs; LASSO, least absolute shrinkage and selection operator; OS, overall survival; qRT-PCR, quantitative real-time polymerase chain reaction; ROC, receiver operating characteristic; TCGA, The Cancer Genome Atlas; ICB, Immune Checkpoint Blockade; TIME, Tumor Immune Microenvironment; TMB, Tumor Mutation Burden; MAF, Mutation Annotation Format; MsigDB, Molecular Signatures Database; TIMER, Tumor Immune Estimation Resource.
