# Supplementary figures and images for: Identification of Prognostic Glycolysis-Related lncRNA Signature in Tumor Immune Microenvironment of Hepatocellular Carcinoma
Source: Front Mol Biosci. 2021 Apr 22;8:645084. doi: 10.3389/fmolb.2021.645084 (PMC8100457; doi:10.3389/fmolb.2021.645084)

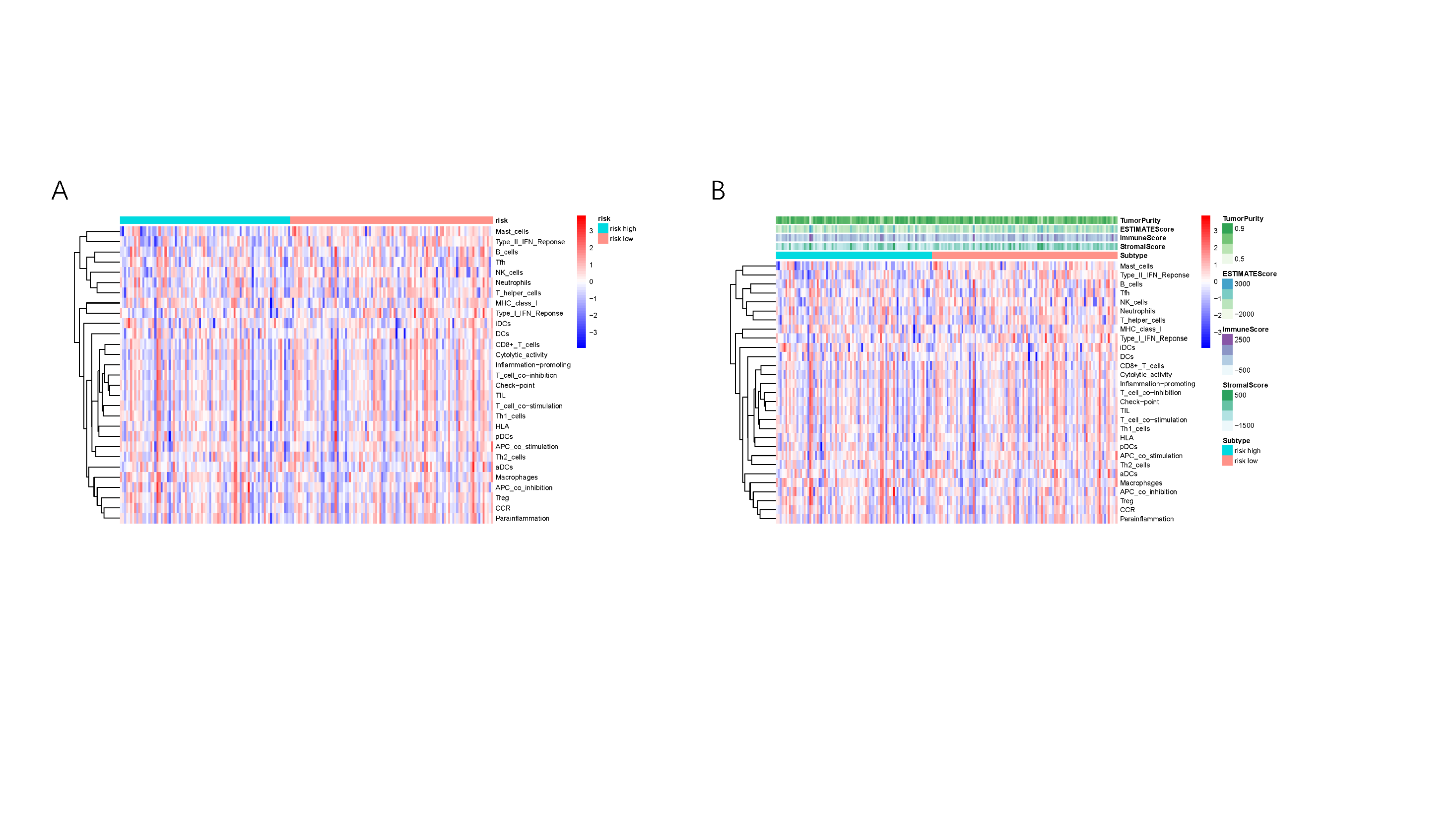

Supplement: Supplementary file 1 [file image3.tiff]

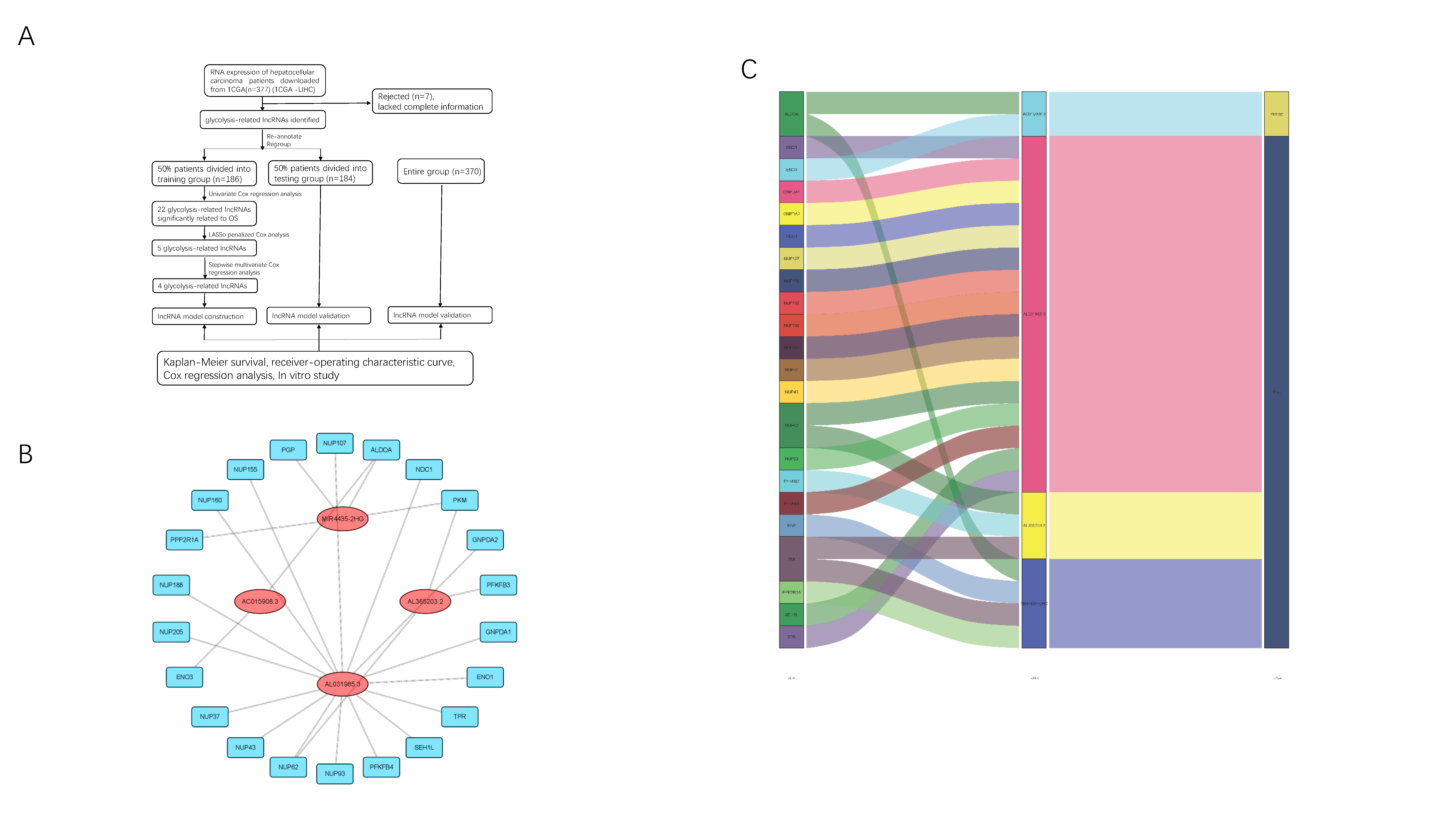

Supplement: Supplementary file 3 [file image1.tiff]

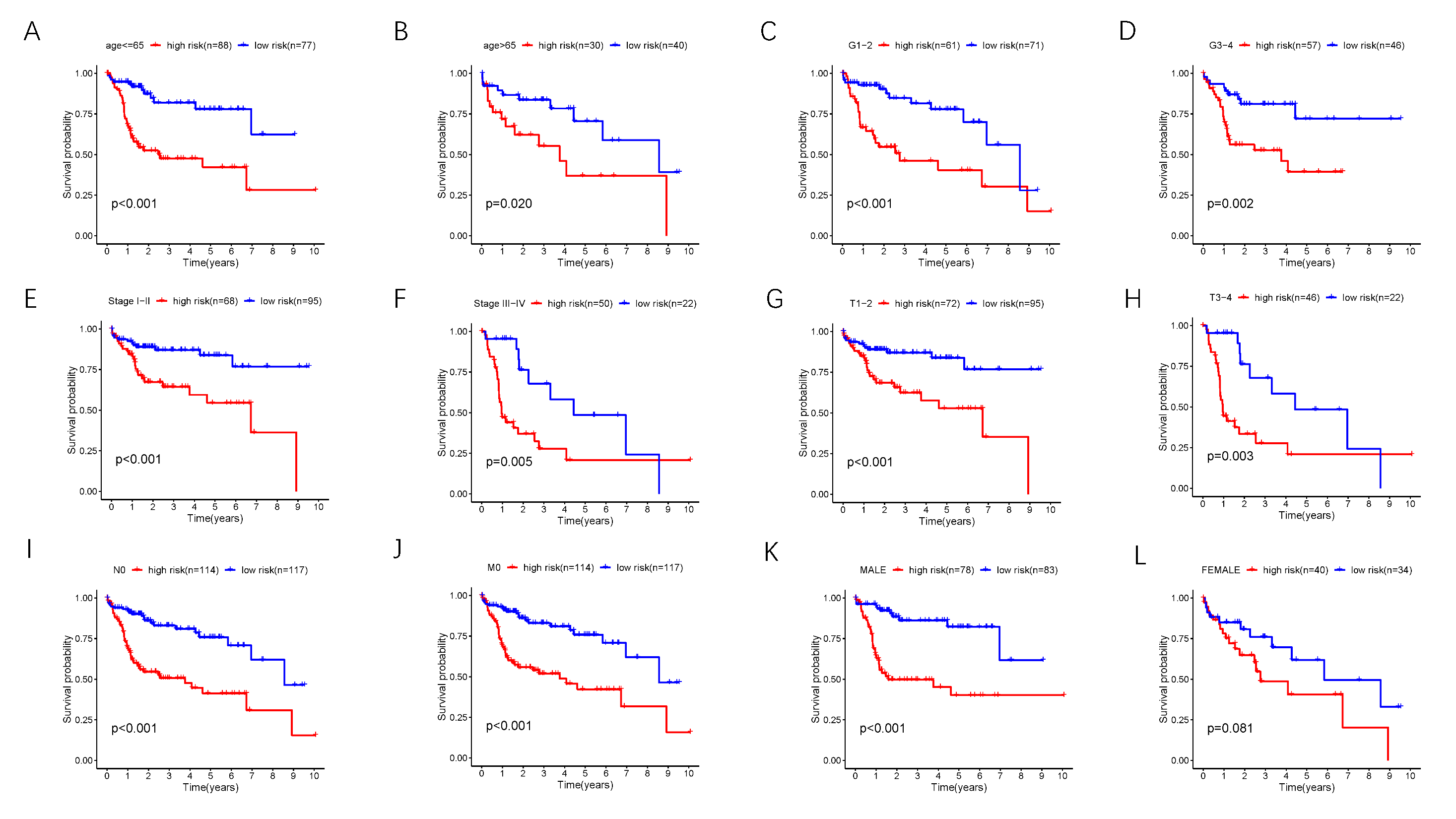

Supplement: Supplementary file 8 [file image5.tiff]

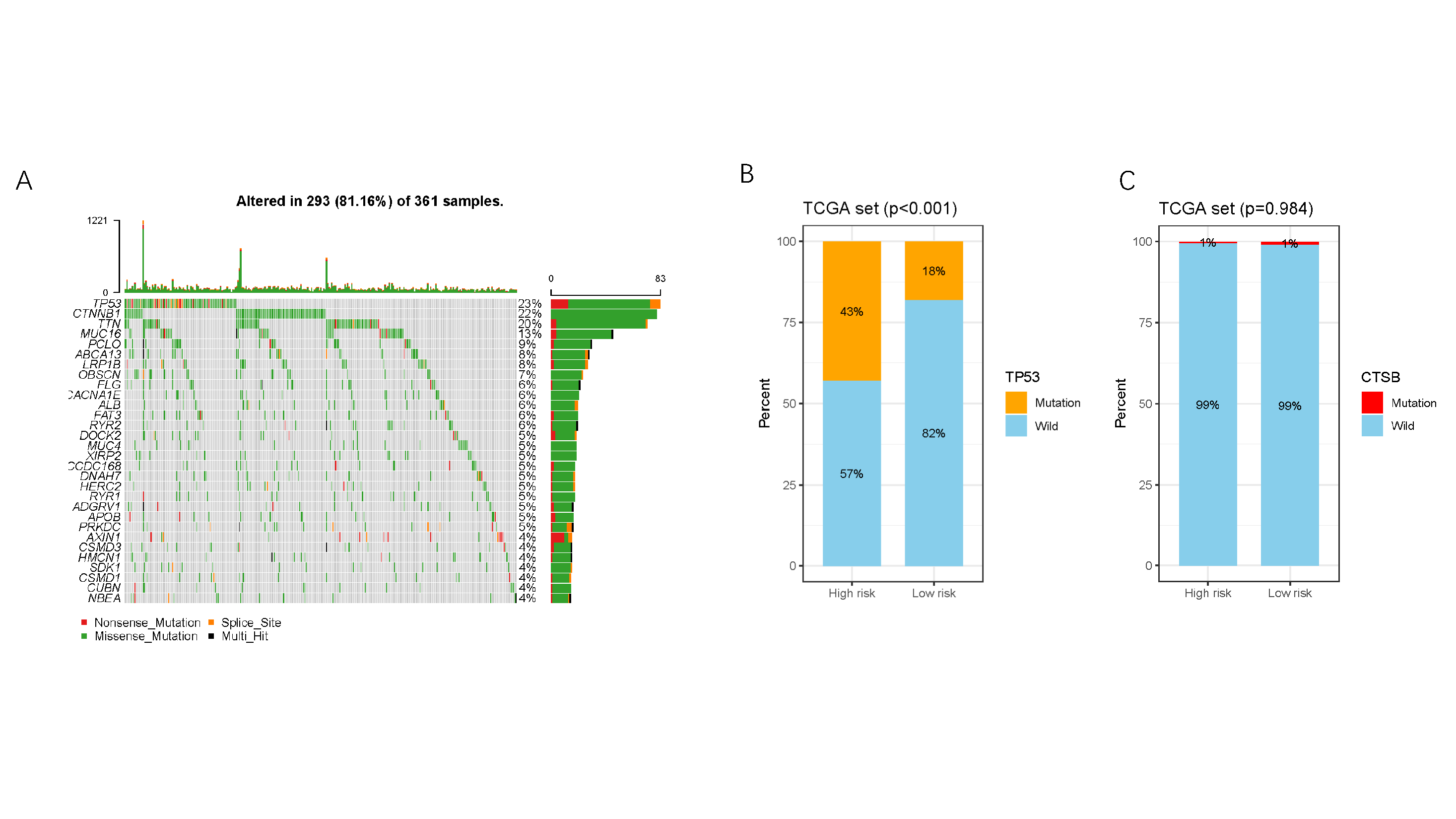

Supplement: Supplementary file 14 [file image6.tiff]

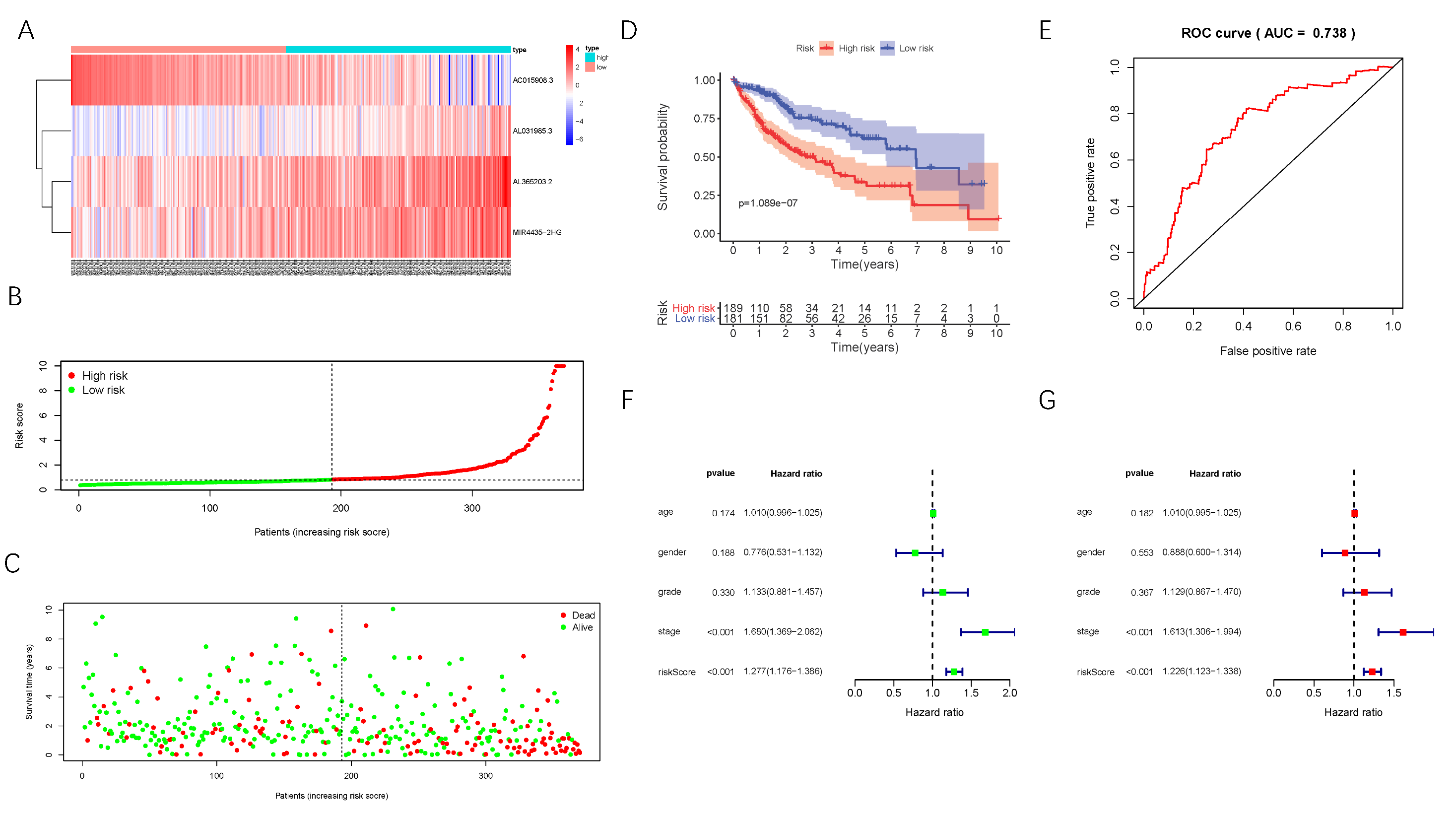

Supplement: Supplementary file 18 [file image2.tiff]

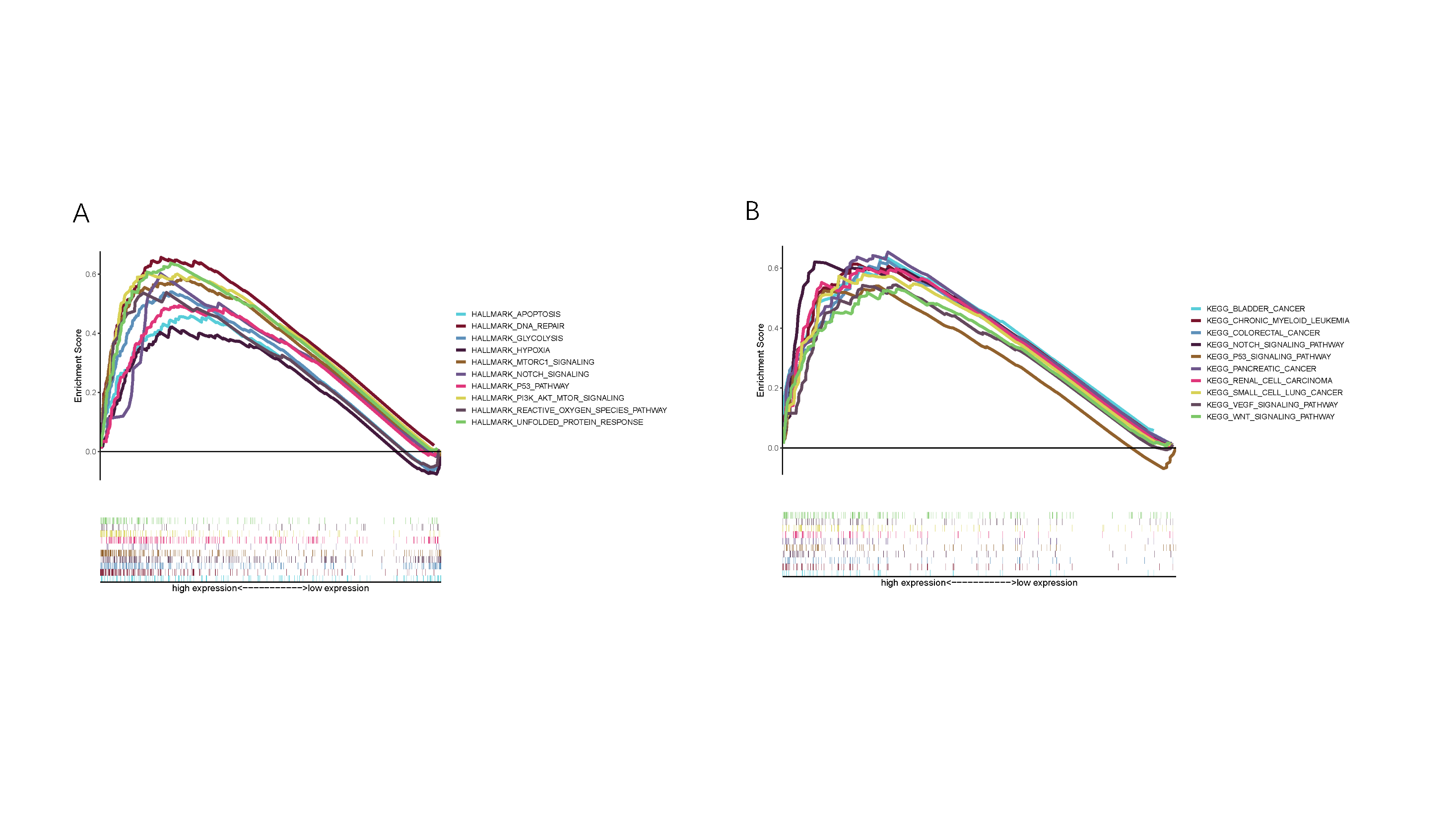

Supplement: Supplementary file 19 [file image4.tiff]
