# Supplementary material for: Identification of Prognostic Glycolysis-Related lncRNA Signature in Tumor Immune Microenvironment of Hepatocellular Carcinoma
Source: Front Mol Biosci. 2021 Apr 22;8:645084. doi: 10.3389/fmolb.2021.645084 (PMC8100457; doi:10.3389/fmolb.2021.645084)
Supplement: Supplementary file 5 [file table1.docx]

**TABLE 1 | Baseline data of all HCC patients.**

| **Characteristic** | **Type** | **n** | **Proportion(%)** |
| --- | --- | --- | --- |
| **Age** | <=65 | 235 | 62.33% |
|  | >65 | 141 | 37.40% |
|  | unknow | 1 | 0.27% |
| **Gender** | FEMALE | 122 | 32.36% |
|  | MALE | 255 | 67.64% |
| **Grade** | G1-2 | 235 | 62.33% |
|  | G3-4 | 137 | 36.34% |
|  | unknow | 5 | 1.33% |
| **Stage** | Stage I-II | 262 | 69.50% |
|  | Stage III-IV | 91 | 24.14% |
|  | unknow | 24 | 6.37% |
| **T stage** | T1-2 | 280 | 74.27% |
|  | T3-4 | 94 | 24.93% |
|  | unknow | 3 | 0.80% |
| **M stage** | M0 | 272 | 72.15% |
|  | M1 | 4 | 1.06% |
|  | unknow | 101 | 26.79% |
| **N stage** | N0 | 257 | 68.17% |
|  | N1 | 4 | 1.06% |
|  | unknow | 116 | 30.77% |
