# Supplementary material for: Identification of Prognostic Glycolysis-Related lncRNA Signature in Tumor Immune Microenvironment of Hepatocellular Carcinoma
Source: Front Mol Biosci. 2021 Apr 22;8:645084. doi: 10.3389/fmolb.2021.645084 (PMC8100457; doi:10.3389/fmolb.2021.645084)
Supplement: Supplementary file 6 [file table15.docx]

**Supplementary Figure Legends:**

**Figure S1:** A) Overall research design. Flow-process diagram presenting the process utilized to identify immune-lncRNAs. The coexpression network and Sankey diagram of prognostic immune-related lncRNAs. B) The coexpression network between prognostic lncRNAs and immune-related genes in HCC. Red round nodes represent prognostic lncRNAs, and the blue round nodes represent immune-related genes. The coexpression network was visualized using Cytoscape 3.7.2 software. C) Sankey diagram showed the association between prognostic immune-related lncRNAs, immune-related genes, and risk types.

**Figure S2:** Verification of the lncRNA signature for predicting HCC prognosis in the entire cohort. A) Heatmap of the 4 glycolysis‐related lncRNAs expression in HCC. The color from green to red shows a trend from low expression to high expression. B) Distribution of lncRNA risk score. C) The survival status and duration of HCC cases. D) Kaplan–Meier curve showing OS in the low- and high-risk groups classified based on the median risk score. E) ROC analysis of the risk scores for overall survival prediction. The AUC was calculated for ROC curves, and sensitivity and specificity were calculated to assess score performance. F) Univariate Cox regression analyses of OS. G) Multivariate Cox regression analyses of OS.

**Figure S3: Correlation of prognostic risk score with immune-related signatures of HCC.** (A)Heatmap displayed enrichment of 29 immune signatures of low-/high-risk groups. Blue represents low activity and red represent high activity. (B)Heatmap of 29 immune signatures and immune scores of two different risk score clusters. Blue represents low activity and red represent high activity.

**Figure S4:** GSEA delineation of the biological pathways associated with the risk scores of this lncRNA signature utilizing the gene set “h.all.v7.2.symbols.gmt [cancer hallmarks]” (A) and “c2.cp.kegg.v7.2.symbols.gmt [Curated]” (B).

**Figure S5: Kaplan–Meier survival analysis for multiple HCC subgroups according to the lncRNAs‐based risk signature stratified by clinical variables.** (A-B) Age. (C-D) Clinical grade. (E-F) Stage. (G-H) T status. (I) N status. (J) M status. (K-L) Gender.

**Figure S6: Correlation of mutation of TP53 with risk score.** (A) Mutation information of each gene in each sample was shown in the waterfall plot, where different colors with specific annotations at the bottom meant the various mutation types. The barplot above the legend exhibited the number of mutation burden. (B) Proportion of mutation of TP53 in both low-/high-risk group form TCGA set. (C) Proportion of mutation of CTSB in both low-/high-risk group form TCGA set.
