# Supplementary material for: Identification of Prognostic Glycolysis-Related lncRNA Signature in Tumor Immune Microenvironment of Hepatocellular Carcinoma
Source: Front Mol Biosci. 2021 Apr 22;8:645084. doi: 10.3389/fmolb.2021.645084 (PMC8100457; doi:10.3389/fmolb.2021.645084)
Supplement: Supplementary file 11 [file table2.docx]

Table 2: Multivariate Cox results of lncRNAs based on TCGA LIHC data.

| id | coef | HR | | HR.95L | HR.95H | pvalue |
| --- | --- | --- | --- | --- | --- | --- |
| AL031985.3 | 0.299987 | | 1.349841 | 0.991382 | 1.837909 | 0.05678 |
| AL365203.2 | 0.105369 | | 1.111121 | 0.987831 | 1.249799 | 0.079101 |
| `MIR4435-2HG` | 0.107428 | | 1.113411 | 0.979232 | 1.265977 | 0.101078 |
| AC015908.3 | -0.25568 | | 0.774388 | 0.595609 | 1.006829 | 0.056244 |
